# Isotope signatures of N_2_O emitted from vegetable soil: Ammonia oxidation drives N_2_O production in NH_4_^+^-fertilized soil of North China

**DOI:** 10.1038/srep29257

**Published:** 2016-07-08

**Authors:** Wei Zhang, Yuzhong Li, Chunying Xu, Qiaozhen Li, Wei Lin

**Affiliations:** 1 Institute of Environment and Sustainable Development in Agriculture, Chinese Academy of Agricultural Sciences, Beijing 100081, China; 2 Environmental Stable Isotope Lab, Chinese Academy of Agricultural Sciences, Beijing 100081, China

## Abstract

Nitrous oxide (N_2_O) is a potent greenhouse gas. In North China, vegetable fields are amended with high levels of N fertilizer and irrigation water, which causes massive N_2_O flux. The aim of this study was to determine the contribution of microbial processes to N_2_O production and characterize isotopic signature effects on N_2_O source partitioning. We conducted a microcosm study that combined naturally abundant isotopologues and gas inhibitor techniques to analyze N_2_O flux and its isotopomer signatures [δ^15^N^bulk^, δ^18^O, and SP (intramolecular ^15^N site preference)] that emitted from vegetable soil after the addition of NH_4_^+^ fertilizers. The results show that ammonia oxidation is the predominant process under high water content (70% water-filled pore space), and nitrifier denitrification contribution increases with increasing N content. δ^15^N^bulk^ and δ^18^O of N_2_O may not provide information about microbial processes due to great shifts in precursor signatures and atom exchange, especially for soil treated with NH_4_^+^ fertilizer. SP and associated two end-member mixing model are useful to distinguish N_2_O source and contribution. Further work is needed to explore isotopomer signature stability to improve N_2_O microbial process identification.

Nitrous oxide (N_2_O) is a greenhouse gas that contributes approximately 6% of the global greenhouse effect and is a major destroyer of the stratosphere^1^,^2^. N_2_O concentration was reported to increase from 270 parts per billion by volume (ppbv) during the pre-industrial period to 327 ppbv currently^3^, and is project to continuously increase during the next few decades^4^. Natural and anthropogenic emissions are the major sources of N_2_O, and approximately 58% of anthropogenic N_2_O emissions are related to agricultural practices around the globe^5^. Among all agricultural activities, cultivation of vegetable crops requires frequent tillage and substantial fertilizer and water, which results in greater production of N_2_O and the emission is not well understood^6^. The area under cultivation for vegetable crops is expanding, and soil in these fields is becoming an immense source of anthropogenic N_2_O production. It is necessary to determine N_2_O sources and the partitioning of individual contributions to total N_2_O emissions to formulate and implement efficient mitigation strategies in agricultural practice. Work related to these critical questions is underway^7^,^8^,^9^,^10^. To our knowledge, there are at least three main N_2_O source processes^11^: (i) nitrification (NN), which is the oxidation of hydroxylamine (NH_2_OH) to nitrite (NO_2_^−^), and includes autotrophic nitrification (AN) and heterotrophic nitrification (HN); (ii) nitrifier denitrification (ND), which is the reduction of NO_2_^−^ by ammonia-oxidizing bacteria; and (iii) denitrification (DD), which is N_2_O production by denitrifiers. These processes may occur individually or in combination in one ecosystem due to the existence of a plethora of diverse microorganisms.

Urea and (NH_4_)_2_SO_4_ are widely used nitrogen fertilizers in agricultural practice; the latter is reported to produce less N_2_O than that produced by urea^12^. NH_4_^+^-N is a substrate for NH_3_ oxidation that can trigger nitrification and nitrifier denitrification^9^ and promote further denitrification^7^, since the NO_3_^−^-N enriched from nitrification is the substrate of the denitrification process. In this study, we used (NH_4_)_2_SO_4_ as N fertilizer with two application levels according to local practice of 100 and 300 mg·N·kg^−1^ dry soil. We hypothesized that different fertilizer amounts could promote different N_2_O production processes and affect their respective contributions to total N_2_O.

Several validated strategies can be utilized to measure N_2_O production and source partitioning, including acetylene (C_2_H_2_) inhibition method^13^, single-label ^15^N method^14^, dual-label ^15^N-^18^O isotope method^9^, and natural abundance isotope technique^15^. Here, we combined the acetylene inhibition method and the natural abundance isotope technique to investigate N_2_O flux and production processes in vegetable soil. We also evaluated the reliability of isotopic signatures [i.e., δ^15^N, δ^18^O and SP (intramolecular ^15^N site preference)] in N_2_O source identification by comparing the results obtained from the two approaches, and provide observations for the related field study.

Here, we report an incubation experiment to determine the effects of fertilizer content in a Chinese cabbage field on N_2_O emissions. The aim was to explore N_2_O emissions and sources in vegetable production, which has not been sufficiently elucidated. The results from this study can facilitate the design of reasonable agricultural mitigation strategies to alleviate the global greenhouse effect.

## Methods

### Soil sampling

Soil (0–20 cm depth) was collected randomly on October 20, 2014, from 10 spots in a field that was planted with Chinese cabbage at the environmental research station of the Chinese Academy of Agricultural Sciences, Shunyi District, Beijing, China (40°15′ N, 116°55′ E). The field had been treated with approximately 400 kg·N·ha^−1^ (equals 150 mg·N·kg^−1^ dry soil) of (NH_4_)_2_SO_4_ for two years. The soil was classified as calcareous Fluvo-aquic according to the Food and Agriculture Organization (FAO). Soil properties at this site were 28.7% sand, 64.2% silt, 7.1% clay, 1.40 g cm^−3^ bulk density, 1.2 g kg^−1^ total N, 13.5 g kg^−1^ organic C, and pH 7.4 (1:2.5, soil/water). Fresh soil was sampled randomly, homogenized, visible roots and other residues were removed, sieved to 2 mm, and refrigerated at 4 °C until use within three days. Soil samples were air-dried for 24 h one day before the start of incubation to eliminate residual N^16^. The soil contained approximately 1.3 mg NH_4_^+^-N per kg dry soil and 30 mg NO_3_^−^-N per kg dry soil before incubation, which was quite low and had little influence on the fertilizer level.

### Experimental setup

A soil microcosm setup was established using the gas inhibitor method to simulate different N_2_O pathways. On day 0, soil was amended with (NH_4_)_2_SO_4_ and deionized water, and homogenized very well to attain 100 (low-dose fertilizer application, group A) and 300 (high-dose fertilizer application, group B) mg·N·kg^−1^ dry soil fertilizer levels and water content of 70%WFPS (water-filled pore space, the initial water content was measured in advance). For each gas treatment, there were six 500-ml glass jars equipped with gas-tight lids and three-way stopcocks. Three of these were for gas analysis, and three were used for soil sampling.

To quantify the contributions of AN, HN, DD, and ND processes in N_2_O emission, six gases were chosen as inhibitors and injected into the jars (Table 1): (i) CK (atmosphere), (ii) N (pure N_2_, purity 99.995%), (iii) O (pure O_2_, purity 99.99%), (iv) LA (air + 0.1% v/v C_2_H_2_, purity 99.6%), (v) HA (pure N_2_ + 10% C_2_H_2_), and (vi) OA (pure O_2_ + 0.1% C_2_H_2_). After loading 100 g of soil to the jars, they were sealed, vacuumed thoroughly, purged three times with the corresponding pure gas, injected with pure or mixed gas (pure gas was injected first, then withdrawn, and replaced with C_2_H_2_), and then incubated in the dark at 25 °C. During the incubation period, soil water content was held constant by checking every 2–3 days using the gravimetrical method. The treatments are named as CK, N, O, LA, HA and OA according to relevant gas inhibitor, and followed by -A or -B which represented treatments in group A and B, respectively.

Gas samples produced during 10.00–12.00 hr on day 1, 2, 3, 5, 7, 10, 15, and 21were collected. On each sampling day, 25 ml of headspace gas was mixed three times with a gas-tight syringe fitted with a pushbutton valve, and then withdrawn to a pre-evacuated gas-tight rubber stopper vial for N_2_O analysis within two days. After gas sampling, all jars were renewed with the respective gas to ensure that incubation conditions remained the same as before sampling. Before measuring the LA, HA, and OA treatments, acetylene was removed with sulfuric acid and potassium permanganate according to the protocol of Malone *et al*.^17^. Then, 10 g of soil was sampled immediately after gas collection from three soil-tested jars for mineral NH_4_^+^-N and NO_3_^−^-N extractions using 50 ml of 2 M KCl, and then stored at −20 °C until colorimetric analysis with a continuous flow analyzer (Futura, Alliance Instruments, France). Ammonium and nitrate were measured using the indophenol blue and sulfanilamide-naphthylethylenediamine methods^18^, respectively.

### Determination of N_2_O flux and isotopic signatures of N_2_O

We measured N_2_O concentrations using an isotope ratio mass spectrometer (IRMS, Isoprime100, Isoprime, Cheadle, UK). Peak area *m*/*z* 44 was used to determine N_2_O concentration with the help of instant atmosphere N_2_O peak area and the published global average N_2_O concentration (327 ppbv)^3^. Pure N_2_ (99.995%) and N_2_O (99.999%) were used as carrier and reference gases, respectively. Calibration was conducted by measuring standards of USGS32 and USGS34. Typical analytical precision was 0.5, 0.9, and 0.6‰ for δ^15^N^bulk^, δ^15^N^α^, and δ^18^O, respectively. The detection limit for N_2_O-N was 500 ppbv. Daily N_2_O flux (μg·N·kg^−1^·d^−1^) was calculated as follows:


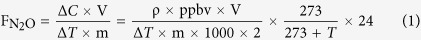


where
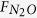
(μg·N·kg^−1^·d^−1^) is N_2_O flux; 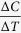
(mg·m^−3^·h^−1^) denotes the rate of increase of N_2_O concentration inside the jar within 2 h (10.00–12.00 hr); ρ (1.964 kg·m^−3^) is N_2_O density at 101.325 kPa and 273 K; V (ml) refers to the headspace volume in the jar (417 and 400 ml for 100 and 300 mg·N·kg^−1^ dry soil, respectively); m (g) denotes the mass of converted dry soil; T (°C) is 25 °C; and 24 is the number of hours within one day. Cumulative N_2_O flux during the experimental period was estimated by averaging the fluxes of two successive determinations, multiplying that average flux by the length of the period between the measurements, and adding that amount to the previous cumulative total. N_2_O isotopomer signatures were determined using the IRMS described above. The isotopic compositions of ^15^N and ^18^O in N_2_O were expressed in δ notation with respect to the atmospheric N_2_ and Vienna standard mean ocean water (V-SMOW), respectively.

Some Equations describing isotopomer ratios of a sample (*R*_sample_) that deviate from ^15^N/^14^N and ^18^O/^16^O ratios of the standard materials (*R*_standard_) are shown below.


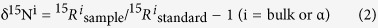



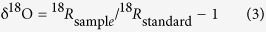



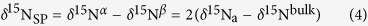


### Statistical analysis

The possible N_2_O production pathways are shown in Table 1. N_2_O fluxes from these processes were calculated as follows:


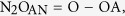



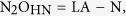



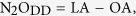



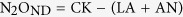


Here, CK, N, O, HA, LA, and OA represent the cumulative N_2_O of their respective gas treatments.

Statistical analyses were performed using Microsoft Excel 2010 and SAS version 9.2 software packages. Significant differences were determined using one-way analysis of variance (ANOVA) and the least significant difference (LSD) test at a 5% level.

## Results

### N_2_O emissions in soil microcosms

**Figure 1:**
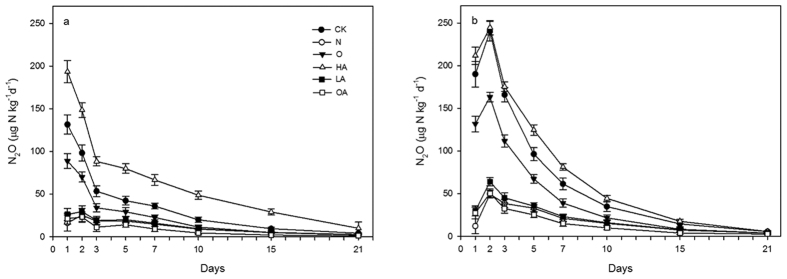
Time series of N_2_O fluxes of treatments in group A (**a**) and B (**b**). Error bars represent standard deviation of the mean (*n* = 3).

Figure 1 presents the time series of N_2_O flux that results from treatment with different gas inhibitors in the low-dose fertilizer group A and the high-dose fertilizer group B. In group A, the peak N_2_O flux occurred immediately after the start of incubation. The greatest flux was measured within 24 h for the HA-A treatment (193.33 μg·N·kg^−1^·d^−1^), which was approximately 12.01 times higher than the lowest flux measured for the N-A treatment (16.09 μg·N·kg^−1^·d^−1^). Daily N_2_O emission from O-A was significantly higher (*P* = 0.001) than that from OA-A on the first day, which indicated that the AN process was inhibited by the addition of C_2_H_2_. The cumulative N_2_O flux in LA-A on the first day (25.99 μg·N·kg^−1^) was higher than that in N-A (16.09 μg·N·kg^−1^), which can be attributed to low HN levels in LA-A enhancing N_2_O emission, however, this difference was not statistically significant (*P* = 0.12).

In group B, the maximum daily flux occurred on day 2 for all treatments, which was one day later than the maxima in group A. We speculate that although the application of a large amount of NH_4_^+^-N fertilizer to the soil delayed the initial microbial response, the subsequent microbial response became stronger as a result of fertilizer application. The results of comparisons between OA-B and O-B (*P* < 0.001) were the same as the results of comparisons of peaks in group A on day 1, whereas LA-B and N-B were significantly different (*P* < 0.001). The weighted average flux of all treatments from group B were significantly higher than those from group A (*P* < 0.05), except for that of HA (*P* = 0.06), which indicated that N_2_O flux increased when more fertilizer was added to the soil.

After the peak emissions occurred, all treatments displayed sharp reductions in N_2_O emission of 39% (LA-A) to 75% (O-A) in group A and 23% (LA-B) to 71% (O-B) in group B; these reductions occurred around day 7, except for N treatment, which decreased slowly and then subsequently increased at a later time in group B. However, N_2_O emission continuously decreased in all treatments, and was depleted at the end of the incubation period.

### Contributions of N_2_O production processes

**Figure 2:**
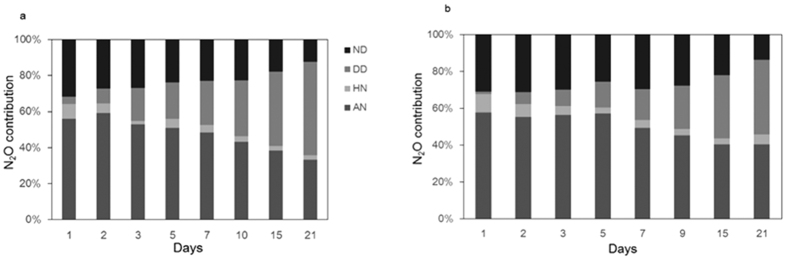
Relative contribution of individual processes to N_2_O production in group A (**a**) and B (**b**) during the incubation period.

In group A, the relative contribution of the AN process increased continuously during the first 5 days and then decreased to 33% at the end of the experiment, which produced 232.23 μg·N·kg^−1^ N_2_O and accounted for 51% of the total N_2_O produced (Fig. 2a). N_2_O emissions from ND peaked on the first day (38.10 μg·N·kg^−1^), which accounted for 32% of the total N_2_O emission on the first day, and then leveled off at 23–27% of the daily total N_2_O emission before declining to approximately 15% of the daily total N_2_O emission during the last two days of the experiment. By contrast, the contribution of DD to N_2_O emissions was weak in the beginning, increased from the third day, and peaked on the last day to account for 52% of total N_2_O emissions, with a weighted average of 18% of total N_2_O emission during the whole period of the experiment. HN remained a weak contributor of 2–8% of N_2_O emission during 21 days, and contributed 5% of the total N_2_O emission.

In group B (Fig. 2b), AN contributed 58% of N_2_O on day 1, and was a significant contributor during the entire experimental period. The cumulative N_2_O emission (258.07 μg·N·kg^−1^) produced by ND in group B was significantly higher than that in group A (*P* < 0.001) and contributed a higher percentage of the total N_2_O compared with that in group A. The daily contribution from DD increased from day 5 and reached the maximum of 41% on the last day of the experiment, which was lower than the observed maximum in group A. However, the cumulative N_2_O emissions produced by AN, HN, ND, and DD processes in soil treated with high-dose fertilizer were significantly higher than those produced in soil treated with low-dose fertilizer (*P* < 0.001). In both group A and B, the AN process was dominant compared with other pathways, followed by the ND process, and then the DD process. Although the DD process only contributed a small proportion the total N_2_O, this contribution increased significantly during the later period of the experiment.

### Variation of mineral N in tested soil

**Figure 3:**
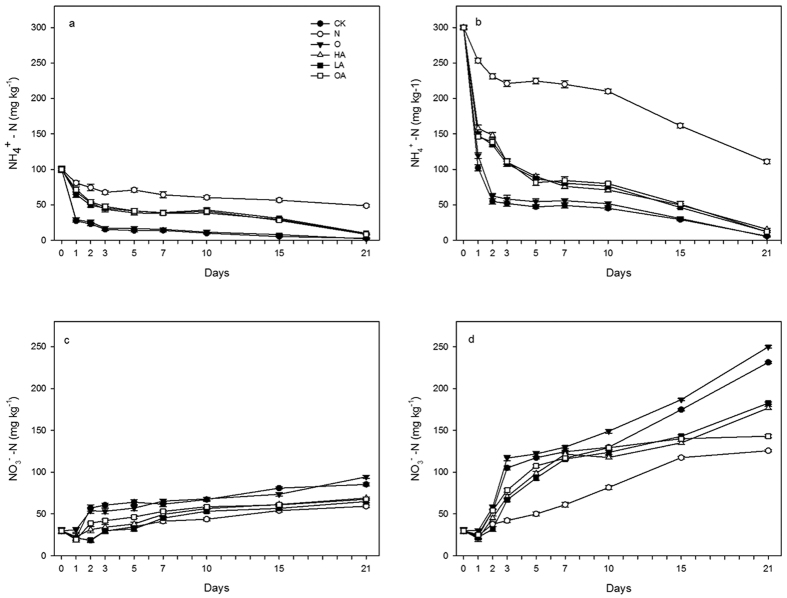
Time series of soil mineral N contents in different treatments. Soil ammonium concentration in group A (**a**) and B (**b**); nitrate concentration in group A (**c**) and B (**d**). Error bars represent standard deviation of the mean (*n* = 3).

The soil concentrations of NH_4_^+^-N and NO_3_^−^-N were analyzed to evaluate possible N_2_O production processes (Fig. 3). The results showed that soil NH_4_^+^-N was rapidly nitrified to NO_3_^−^-N after the start of incubation. In the CK-A and O-A treatments, NH_4_^+^-N drastically decreased by 73% within 24 h, which indicated that NN and ND might be the main processes in these treatments. By contrast, NH_4_^+^-N decreased slowly in the N-A treatment, with a reduction of 51% over the entire incubation period, which suggests that NH_4_^+^-N breakdown might be initiated by other N_2_O production processes or by natural degradation rather than by nitrification. NH_4_^+^-N breakdown in group B treatments also was observed. The period of rapid reduction lasted for 48 h, or one day longer than in group A, which was consistent with the peak N_2_O flux observed in both groups. The weighted average NH_4_^+^-N contents of treatments in group B were significantly different from those in group A (*P* < 0.01), and they were all substantially depleted (90–98%) at the end of the experiment, except for N contents (59–63%) in both groups. NO_3_^−^-N contents increased sharply within 2 days (group A) and 3 days (group B) in all treatments except for LA-A, N-A, and N-B treatments, then increased gradually until reaching the maximum concentrations on day 21 (Fig. 3c,d). In groups A and B, NO_3_^−^-N accumulated rapidly in the O (53.95 and 116.92 mg·kg^−1^) and CK (57.12 and 104.97 mg·kg^−1^) treatments during 48 h and 72 h, respectively, which were consistent with the reduction of NH_4_^+^-N, and indicated that the AN process was predominant in the CK and O treatments. In the LA and OA treatments, the cumulative NO_3_^−^-N contents were between the levels of O and N, which suggests that nitrification consumes NO_3_^−^-N to some extent. In the N treatment, the cumulative NO_3_^−^-N content was less than that in other treatments in both groups, which suggests that DD was the sole process occurring in this tested soil. The variation in N levels indicated that NH_4_^+^-N transformation to NO_3_^−^-N via N_2_O production (NN, ND, and DD processes) occurred in all treatments of both groups except for the HA treatment.

### Measurement of denitrification products ratio N_2_O/(N_2_O + N_2_)

The pathway of N_2_O reduction to N_2_ is important for understanding N_2_O consumption in agricultural soil, and it is a possible target for mitigation of N_2_O emissions. The combination of C_2_H_2_ and N_2_ (v/v = 1:9, HA) inhibits N_2_O reduction to N_2_; therefore, we analyzed N_2_O fluxes produced in CK and HA treatments to estimate the contents of potential denitrification products (N_2_O + N_2_) and the ratio of N_2_O/(N_2_O + N_2_). The results showed that both N_2_O and (N_2_O + N_2_) emissions were significantly higher (*P* = 0.01 and 0.04, respectively) in group B (weighted average daily fluxes of 52.41 and 60.5 μg·N·kg^−1^·d^−1^, respectively) than in group A (weighted average daily fluxes of 26.66 and 50.15 μg·N·kg^−1^·d^−1^, respectively). The weighted average ratios [N_2_O/(N_2_O + N_2_)] were 0.44 and 0.87 in group A and group B, respectively, which were significantly different (*P* = 0.002). The ratio of 0.87 indicates that only 13% of N_2_O was reduced to N_2_, and a substantial percentage of the remainder was lost. N_2_O emissions showed a linear and positive relationship with total denitrification emissions (N_2_O + N_2_) in low-dose (*P* < 0.001, *R*^2^ = 0.99) and high-dose (*P* = 0.005, *R*^2^ = 0.99) fertilizer-amended soil. In group A, the N_2_O and (N_2_O + N_2_) contents were significantly correlated with the ratio of N_2_O/(N_2_O + N_2_) (*P* = 0.009, *R*^2^ = 0.81; *P* < 0.001, *R*^2^ = 0.80, respectively). These relationships were not robust in group B (*P* = 0.007, *R*^2^ = 0.22; *P* = 0.005, *R*^2^ = 0.16, respectively).

### Measured isotopic signatures of N_2_O in soil microcosms

**Figure 4:**
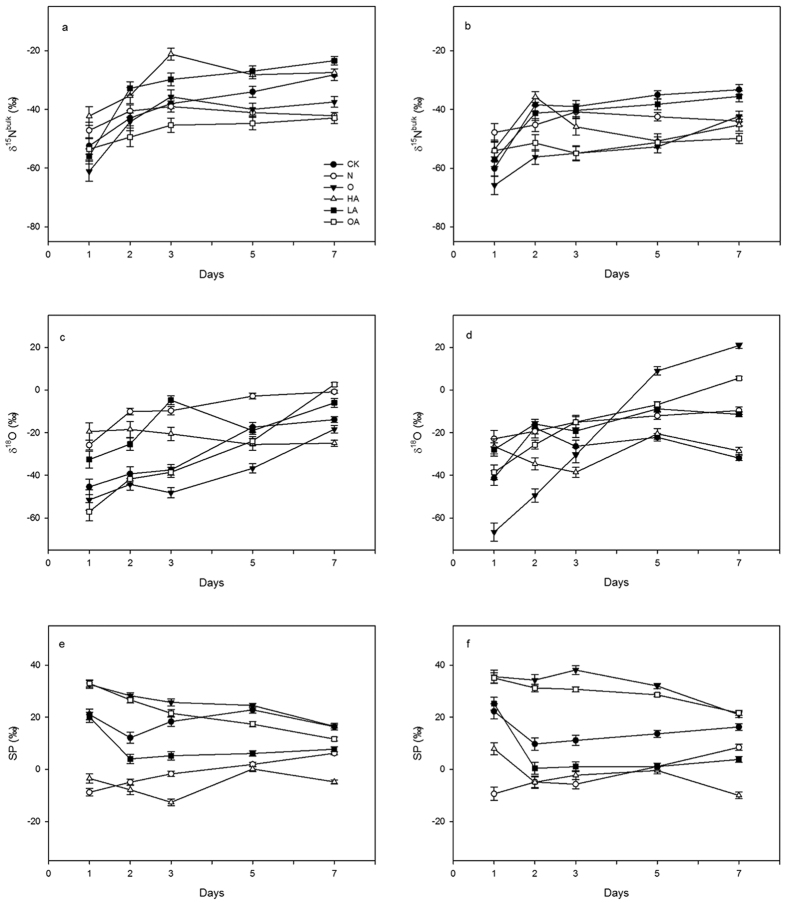
Time series of δ^15^N^bulk^, δ^18^O, and SP of N_2_O in different treatments in group A (**a,c,e**) and B (**b,d,f**). Error bars represent standard deviation of the mean (*n* = 3).

We explored the isotopomer signature profiles during the first week of the incubation period because N_2_O isotopic composition is influenced primarily by the peak flux, and in agronomic applications the peak generally occurs within one week after use^19^. We found that δ^15^N^bulk^ of N_2_O increased during the first week in both groups (Fig. 4a,b), which ranged from −65.85‰ (the lowest) on the first day to −27.43‰ (the highest) on day 7. This is in agreement with previous studies, which report that δ^15^N^bulk^ of N_2_O usually increases after urea^20^ or ammonia fertilizer^21^ application. The weighted average of δ^15^N^bulk^ of each treatment in group B was lower than those of each treatment in group A, and this is consistent with previous studies^19^,^20^ showing that N_2_O emissions increase as fertilizer content increases, whereas the δ^15^N^bulk^ value was reduced as the fertilizer content increased.

The time gradient (Δδ^15^Ν^bulk^/ΔΤ) of ^15^N^bulk^ was analyzed because the changes in ^15^N^bulk^ after addition of NH_4_^+^ might complicate the use of ^15^N^bulk^ to identify N_2_O source partitioning. In both groups A and B, the ^15^N^bulk^ of the CK and O treatments had relatively high time gradients ranging from 3.36 to 3.46‰ d^−1^, which were consistent with the reduction of NH_4_^+^-N concentration in the tested soil. Accordingly, we assumed that the highest gradient should be from the O treatment rather than the CK treatment. The reason for this bias might be the reduction of N_2_O to N_2_ in the CK treatment, which generally leads to an increase in the value of δ^15^N^bulk ^15,19. The minimum time gradient for appearance of δ^15^N^bulk^ came from the N in both groups A (0.71‰ d^−1^) and B (0.55‰ d^−1^) This might due to the relatively low fractions of consumed NH_4_^+^ because, theoretically, nitrification would not occur. The observed slight increase in δ^15^N^bulk^ of OA suggested the occurrence of weak HN. A comparison of temporal parameters indicates that shifting of δ^15^N^bulk^ is related to the fraction of NH_4_^+^ consumption (e.g., nitrification requires more NH_4_^+^-N, whereas denitrification does not). These considerations helped us to identify the candidate processes that occurred in these treatments.

It is more complicated to describe the variation of δ^18^O-N_2_O than that of δ^15^N^bulk^, because the O atom readily exchanges with O_2_, H_2_O, and substrate NO_3_^−^. In this study, neither a stable variation nor significant differences (*P* > 0.05) between groups A and B were observed (Fig. 4c,d). Most δ^18^O values were lower than measured instant values of δ^18^O-H_2_O (−1.4‰) and δ^18^O-O_2_ (19.7‰), but were close to another incubation experiment we performed that used the same soil and fertilizer. δ^18^O increased rapidly during the first week in the O and OA treatments, which might be due to substantial nitrification promoting O-exchange with H_2_O and O_2_, and thus resulting in the enrichment of ^18^O in the remaining O atom. In the CK and LA treatments, denitrification drives NO_3_^−^ consumption and enrichment of ^18^O in the remaining NO_3_^−^ in the soil, which increases δ^18^O abundance. It was reported that cleavage of the N-O bonds during N_2_O reduction by denitrification would lead to the accumulations of both δ^15^N^α^ and δ^18^O in the residual N_2_O^19^,^22^,^23^,^24^. This might explain why δ^18^O from HA fluctuated slightly, as denitrification was believed to be the sole process and reduction was not believed to occur. However, ^18^O is not suitable for N_2_O partitioning because O atoms exchange frequently with different O sources and the O sources vary with respect to δ^18^O.

### Evaluation of N_2_O source partition on SP

The weighted average SP in the CK-A and CK-B treatments were 18.58‰ and 14.71‰, respectively, which was higher than that reported for denitrification (−10 to 0‰) and lower than that reported for nitrification (33 to 37‰) from pure culture experiments^25^ (Fig. 4e,f). This indicated that multiple N_2_O processes occurred simultaneously in the CK treatment. The SP of CK and LA treatments of both groups decreased sharply on day 2, and then increased until day 7. This result might suggest that N_2_O reduction occurred after the mixing of produced N_2_O, which is consistent with the results of N_2_O emission in a closed system. In the N treatment, SP increased gradually and reached 6.22‰ and 8.46‰ in group A and B, respectively, on day 7. This result indicated that fungal denitrification might occur, because its average SP value is 30.0 ± 4.8‰, according to Maeda *et al*.^26^. In the O treatment, SP slightly fluctuated during the first 5 days, and then decreased to 16.49‰ and 21.0‰ in group A and B, respectively, on day 7. We inferred that aerobic denitrification induced by microbes such as *Pseudomonas* spp. and *Alcaligenes* spp. occurred, thereby lowering SP values in the system. Similarly, the successive decline of SP in OA and LA treatments might be attributed to the HN process accompanied by aerobic or anaerobic denitrification, respectively. For the HA treatment, SP matched the range of −10 to 0‰ during the whole week, except for HA-B on day 1, which indicated that DD was the single process occurring. Meanwhile, no obvious increase in SP was observed, which is consistent with the conclusion that N_2_O reduction would be inhibited by high C_2_H_2_ concentration.

In this study, the two end-members mixing model [Eq. (5)]^27^ was applied to evaluate the respective contributions of pairs of processes in the CK treatment on day 1 and 7. Four cases should be considered depending on higher-SP and lower-SP combinations^28^: (i) NN_bacteria_ and DD_baceria_, (ii) NN_bacteria_ and ND_bacteria_, (iii) DD_fungus_ and DD_bacteria_, and (iv) DD_fungus_ and ND_bacteria_. To simplify the model, we only discuss cases 1 and 2 for CK treatment. For example, the contribution of nitrification in case 1 can be expressed as shown below. The isotopomer signatures and possible contributions of processes are presented in Table 2.


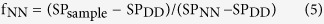


In Eq. (5), f_NN_ is the contribution of N_2_O derived from nitrification; SP_sample_ is the measured SP value of N_2_O; and SP_NN_ (33‰), SP_DD_ (−10 to 0‰), and SP_ND_ (−13.6 to 5‰) represent the respective SP values of nitrification, denitrification, and nitrifier-denitrification processes reported by previous studies^25^,^28^.

The respective contributions of NN (AN + HN), DD, and ND estimated by N_2_O measurement were consistent with the calculated ranges based on model cases 1 and 2. N_2_O flux from DD was relatively weak during the first week, suggesting that NN and ND were more likely to be involved in the CK treatment. This was confirmed by the comparison between the model prediction and N_2_O measurement. We observed that ND contributions calculated from the model were higher than those of actual measurements in groups A and B on day 7, which indicates that they were overestimated by the model. This can be attributed to the observation that the contribution from DD became more prominent at later time points, which was not accounted for by the two end-members model system.

## Discussion

Previous studies have investigated N_2_O emission pathways in agricultural soil treated with ammonia fertilizer^14^,^29^,^30^. N_2_O production and consumption in soil is generally mediated by microbes and microbial communities, which metabolize soil nutrients, regulate N_2_O emissions and sources, and determine the contributions of different processes to total N_2_O emissions in different ecosystems. The studies agree that sources of N_2_O emissions vary as environmental factors change. In the current study, autotrophic nitrification was a prominent pathway generating N_2_O emissions, whereas heterotrophic nitrification had a minor role in the tested soil. This result is consistent with some previous reports^7^,^14^, but it is not in agreement with other reports^29^,^31^ that observed a large contribution from HN to N_2_O emissions, especially in acidic soil or soil with a high organic carbon content. These inconsistencies may be attributed to heterogeneous soil textures, organic components, and pH. Conversely, the contribution of HN might be underestimated in the current study because C_2_H_2_ inhibits the HN pathway^9^,^14^.

Nitrifier denitrification has been reported as another important source of N_2_O production, and it accounts for 30–66% of N_2_O emissions in pure cultures^32^. In the current study, the contribution from ND increased greatly with higher fertilizer content in the tested soil. This can be explained by extremely high NH_4_^+^ levels, suboxic conditions, and low organic carbon contents, which created conditions that were more favorable for ND than other processes^30^. The low contribution from denitrification observed in this study was related to the absence of initial NO_3_^−^ and the presence of only a few denitrifiers in the soil. The latter condition was verified by subsequent field study results that detected relatively few *nirS* genes but abundant *amoA* genes in the same soil used in the current experiments. The contribution of DD continuously increased and became the main process during the later experimental period due to transformation of NH_4_^+^ to NO_3_^−^. However, the DD contribution was less than that of AN because the total N_2_O flux decreased greatly during the later period. This result indicates that DD has a weak contribution at 70% WFPS. Although this result is not consistent with previous studies, it is supported by several recent analyses of global trends in the ^15^N, ^18^O, and SP signatures of N_2_O, which suggest that ammoniacal N fertilizers and nitrification are the principal sources responsible for the rise in atmospheric N_2_O^9^,^33^.

Other pathways in this study refer to dissimilatory nitrate reduction to ammonium (DNRA), chemodenitrification, and chemonitrification. DNRA has been reported to significantly contribute to N_2_O emissions under certain conditions^34^,^35^. It requires more energy to reduce NO_3_^−^ to NH_4_^+^ and is favorable for conditions with high C:NO_3_^−^ ratio; therefore, it is reasonable to hypothesize that DNRA contributes weakly to N_2_O emissions in our local vegetable soil. Although we cannot track chemodenitrification and chemonitrification patterns in our data, the existence of these processes should not be ignored. Future work should investigate these unusual N_2_O production pathways.

The final step in denitrification is the conversion of by-product N_2_O to N_2_ by nitrous oxide reductase. This step is critical for evaluating N_2_O consumption and to understand nitrogen accumulation in soil and emission to the atmosphere. The ratio of denitrification products [N_2_O/(N_2_O + N_2_)] was used to evaluate the degree of N_2_O conversion to N_2_, which ranged from 0 (all N_2_O was reduced to N_2_) to 1 (N_2_O was the sole terminal denitrification product)^36^. Our results show that the denitrification product ratio in soil with high NH_4_^+^ fertilizer content is significantly higher than that in soil with low NH_4_^+^ fertilizer content (*P* < 0.001), which indicates that the higher the fertilizer content, the larger the ratio of denitrification products and loss of N. It results in more N_2_O gas and NO_3_^−^ production which emit to atmosphere, immobilize in soil, diffuse into groundwater, and cause severe environmental pollution. The soil is a source rather than a sink in this case. Previous work showed that high N_2_O production was generated primarily by NH_3_ oxidation pathways under low O_2_ availability^9^, which is also consistent with our results. This suggests that N_2_O emissions could be reduced by avoiding high ammonium concentrations in the soil, or by selecting a different type of fertilizer to mitigate N_2_O emissions, especially in vegetable fields that require high water content (which causes low oxygen concentration) and fertilizer content.

Some studies reported that δ^15^N^bulk^ is a good indicator for distinguishing nitrification and denitrification because nitrification more strongly depletes ^15^N compared with denitrification^20^,^37^. Other authors contend that δ^15^N^bulk^ can be affected by NH_4_^+^ and NO_3_^−^ origins, microsite heterogeneity, and reductant^7^,^11^. Here, we observed that δ^15^N^bulk^ level increased after NH_4_^+^ application due to isotope fractionation during nitrification, which enriches the remaining ^15^N of N_2_O. Therefore, Δδ^15^N (the difference between δ^15^N of the substrate and the product) was proposed to differentiate among N_2_O sources in N-fertilized agricultural soil, and approximate ranges of different pathways were reported^15^,^37^. In the current study, data for Δδ^15^N varied greatly and was not constant over time due to δ^15^N^bulk^ of the precursor (NH_4_^+^). Therefore, we cannot use this parameter to identify N_2_O sources. We analyzed an alternative time course of δ^15^N^bulk^ and found that it did provide some information regarding possible processes. However, we do not recommend δ^15^N^bulk^ as a powerful indicator for N_2_O source partitioning, especially under the conditions of NH_4_^+^ fertilizer application. The possible process stated in this paper was deduced based on whether a process occurs or not and by estimating possible sources in advance. Currently, there is a lack of robust evidence that identifies a distinct range of δ^15^N^bulk^ associated with different microbial sources. Therefore, N_2_O source partitioning based solely on δ^15^N^bulk^ should be treated with caution, and additional studies are needed. However, it is useful to identify natural and anthropogenic N_2_O sources because δ^15^N^bulk^ of N_2_O emitted from agricultural fields is more depleted than that from N-limited soil^19^, as shown in this study.

The δ^18^O value is not only affected by reduction of N_2_O to N_2_^23^ but also by exchanging of O atom with O_2_, H_2_O, and NO_3_^−^38,39. Previous studies reported that 100% of N_2_O-O is derived from O_2_ during NH_2_OH oxidation. Half of this originates from O_2_ and the other half is from H_2_O from the NO_2_^−^-N_2_O reaction, or completely from substrate NO_3_^−^ during denitrification of NO_3_^−^ fertilizer^38^, or N_2_O-O derived from both O_2_ and NO_3_^−^ during the NO_3_^−^ to N_2_O step. The contributions of H_2_O-O and NO_3_^−^ O to N_2_O-O also are influenced by the species present^39^. Therefore, the original sources of O in N_2_O are hidden and uncertain so that δ^18^O is questionable as a stable indicator in some cases. Although ranges of δ^18^O values for process identification are given in some studies^7^,^40^, it remains debatable and not reliable to use only δ^18^O for source partitioning. A combination with other isotopomer signatures, such as δ^15^N or SP, might improve its accuracy. Compared with δ^15^N^bulk^ and δ^18^O, SP is a powerful tool for N_2_O source partitioning^11^ due to the minimal disturbance for samples^25^ and independence of the precursor δ^15^N composition^37^. However, it is affected by some factors such as microbial genera^26^,^27^, N_2_O reduction to N_2_^24^, and soil heterogeneity^41^. SP values used for source partitioning were measured from pure culture experiments in the laboratory, which significantly differ from microbial communities in natural environments. It is not possible to partition N_2_O sources only by SP value in a complex ecosystem because multiple microbial processes coupled with reduction sometimes occur simultaneously in the soil matrix. Therefore, one of the great challenges is to provide an explicit scope of SP values associated with different microbial processes or their combinations. Future studies should characterize SP, generate data sets, determine standard calibrations, and link to microbial ecology using molecular approaches^11^, such as high-throughput sequencing technique to ascertain the relationships between relevant microbial communities and N_2_O source partition in order to reduce the current discrepancies.

Using the two end-members mixing model, we estimated the approximate contributions of process pairs using the SP values derived in this study. This was a quantitative rather than qualitative analysis. The combinations of NN_bacteria_-ND_bacteria_ and NN_bacteria_-DD_bacteria_ gave reasonable estimates for nitrification contributions, and NN_bacteria_-ND_bacteria_ was closer to the real measured proportion of N_2_O. In both groups A and B, the contributions of nitrification calculated from the model NN_bacteria_-DD_bacteria_ were higher than the measured values. This finding was not in agreement with a previous report that nitrification was usually overestimated by the inhibition approach in the presence of 0.01% C_2_H_2_ compared with the estimation using the ^15^N-^18^O isotope method^9^. One possible reason for this discrepancy is that fungal denitrification occurred and enhanced both SP value and contribution of nitrification, as shown in Table 2. Another possible reason is that all N_2_O emissions are obtrusively grouped into two sources and might overestimate the contribution of a single process. Different SP ranges applied in the model will produce different contributions; currently, there is not a distinct range of SP values for nitrifier denitrification. We estimated its relative contribution using an SP range of −13.6 to 5‰ as reported by Zou *et al*.^28^ to encompass the widest scope of published SP values for nitrifier denitrification, rather than using the SP value of −3.8‰ reported by Frame and Casciotti^27^, −1.0‰ reported by Decock and Six^24^, or −10.7 to 0.1‰ reported by Wunderlin *et al*.^15^. However, the conclusions from SP evaluation and N_2_O measurement methods are similar irrespective of the method.

The measurements and analyses performed in this study demonstrate the possible application of a natural isotope abundance approach to determine N_2_O sources in vegetable soil. The combination of this approach with N_2_O flux measurements in the future could help to verify, quantify, and understand microbial processes within the spatiotemporal scale of the environment.

## Additional Information

**How to cite this article**: Zhang, W. *et al*. Isotope signatures of N_2_O emitted from vegetable soil: Ammonia oxidation drives N_2_O production in NH^4^^+^-fertilized soil of North China. *Sci. Rep.* **6**, 29257; doi: 10.1038/srep29257 (2016).

## Figures and Tables

**Table 1:** Inhibitors used and their effects on N_2_O production processes.

Treatment	AN	HN	DD	ND	Other
CK	+	+	+	+	+
N	−	−	+	−	+
O	+	+	−	−	+
LA	−	+	+	−	+
HA	−	−	+	−	+
OA	−	+	−	−	+

“+” indicates that the process occurs, “−” indicates that the process is blocked (based on previous work^30^,^42^) and slightly modified.

**Table 2:** Isotopomer signatures of N_2_O and contributions of different pathways on N_2_O production.

		δ^15^N (‰)		δ^18^O (‰)		δ^15^Ν^α^ (‰)		SP (‰)		Contribution to N_2_O production based on SP (%)				Contribution to N_2_O production based on measurement (%)	
		Day 1	Day 7	Day 1	Day 7	Day 1	Day 7	Day 1	Day 7	Day 1^a^		Day 7		Day 1	Day 7
										Case 1	Case 2	Case 1	Case 2		
CK	A	−52.44	−28.20	−45.40	−13.86	−41.91	−20.01	21.06	16.38	NN: 64–72 DD: 28–36	NN: .57–74, ND: 26–43	NN: 50–61, DD: 39–50	NN: 41–69, ND: 31–59	AN : 56, HN: 8, DD: 4, ND: 32	AN : 48, HN: 4, DD: 25, ND: 23
	B	−60.10	−33.28	−41.27	−31.91	−48.97	−25.14	22.27	16.26	NN :67–75 DD: 25–33	NN: .62–82, ND: 18–38	NN: 49–61, DD: 39–51	NN: 40–68, ND: 32–60	AN : 58, HN: 10, DD: 2, ND: 31	AN : 49, HN: 4, DD: 17, ND: 29
N	A	−47.17	−42.18	−25.81	−17.80	−51.56	−39.07	−8.79	6.22	DD: 100		DD: 44–55, DD_fungus_: 45–56^b^		DD: 100	nd
	B	−47.81	−43.76	−22.81	−25.61	−52.51	−39.73	−9.39	8.46	DD: 100		DD: 34–43, DD_fungus_: 57–66		DD: 100	nd
O	A	−61.13	−37.47	−51.50	18.36	−44.85	−29.22	32.56	16.49	NN: 100		NN: 50–62, DD: 38–50		NN: 100	nd
	B	−65.85	−42.34	−66.57	20.82	−48.06	−31.84	35.58	21.00	NN: 100		NN: 64–72, DD: 28–36		NN: 100	nd
HA	A	−42.29	−27.43	−19.47	−24.91	−44.03	−29.83	−3.49	−4.80	DD: 100		DD: 100		DD: 100	DD: 100
	B	−53.64	−45.30	−26.44	−28.51	−49.68	−50.30	7.92	−9.99	nd		DD: 100		DD: 100	DD: 100
LA	A	−55.99	−23.41	−32.62	−5.99	−45.99	−19.54	20.00	7.73	HN: 61–70, DD: 30–39		HN: 23–41, DD: 59–77		nd	nd
	B	−57.10	−35.55	−27.92	−11.39	−44.51	−33.65	25.19	3.81	HN: 76–82, DD: 18–26		HN: 12–32, DD: 68–88		nd	nd
OA	A	−53.53	−42.96	−57.05	2.62	−37.05	−37.13	32.96	11.66	HN: 100		HN: 35–50, DD: 50–65		HN: 100	nd
	B	−54.02	−49.86	−38.53	5.54	−36.50	−39.05	35.04	21.63	HN: 100		HN: 66–73, DD: 27–34		HN: 100	nd

^a^NN, DD, ND represent nitrification, denitrification, and nitrifier denitrification processes performed by bacteria.

^b^Contributions of different N_2_O production pathways were based on case 3 of the two end-members mixing model.

nd, not determined with this method.
